# Sexual violence against children and adolescents in Paraná State: geospatial analysis and main socioeconomic indicators

**DOI:** 10.1016/j.jped.2024.03.014

**Published:** 2024-05-01

**Authors:** Carolina Sartini Stocco, Tiago Francisco Meleiro Zubiolo, Matheus Henrique Arruda Beltrame, Cátia Millene Dell'Agnolo

**Affiliations:** a Universidade Estadual de Maringá, Departamento de Medicina, Maringá, PR, Brazil; b Universidade Estadual de Maringá, Grupo de Estudos em Tecnologias Digitais e Geoprocessamento em Saúde (GETS), Maringá, PR, Brazil; c Universidade Estadual de Maringá, Departamento de Medicina, Programa de Pós-Graduação em Gestão, Tecnologia e Inovação em Urgência e Emergência, Maringá, PR, Brazil

**Keywords:** Sex offenses, Child abuse, Child abuse, sexual, Public health surveillance, Abuse notification

## Abstract

**Objective:**

Child sexual violence is a multidimensional problem of many contemporary societies, affecting people of all sexes, social stratum and age groups. Offenses involving children and adolescents are more serious, given their total or partial dependence on parents and caregivers. Information on child sexual violence in Brazil is found in raw form and without detail. The objective was to compare the information with social and economic data in the state of Paraná.

**Methods:**

The authors conducted a retrospective study of secondary data from the Notifiable Diseases Information System (SINAN) on cases of sexual violence involving victims aged 0 to 19 years. Results are presented according to notification characteristics. The authors applied exploratory spatial data analysis to assess spatial autocorrelations and investigated relationships by the ordinary least squares regression model.

**Results:**

Between 2017 and 2021, there were 13,403 reports of child sexual violence in Paraná State, Brazil. Most victims (82.8%) were female and aged between 10 and 14 years. The majority of sexual violence cases (67.8%) occurred in the home environment. The highest rates on a population basis were observed in the North Central and Greater Curitiba regions, mainly in cities with higher population density and with higher rates of other types of violence.

**Conclusion:**

The results provide data that can promote a broader understanding of the distribution of sexual violence and the state and associated variations. It is expected to improve the provision of care for victims of child sexual violence and assist in strategic planning to prevent future offenses.

## Introduction

Sexual violence is a structural problem in many contemporary societies that causes serious physical consequences and emotional trauma to victims and their families.^1^ This multidimensional phenomenon affects people of all sexes, age groups, social strata, ethnic groups, and sexual orientations, constituting one of the main forms of human rights violation and a threat to the right to life, health, and physical integrity, however, women of all ages (mainly teenagers) are the main victims.^2^^,^^3^ Due to this more frequent profile of victims, the authors chose the population aged 0 to 19 to evaluate in this study.

In Brazil, a major step toward the universalization of continuous surveillance occurred in 2008 with the inclusion of sexual violence cases in the Notifiable Diseases Information System (SINAN), a national health information system, under the responsibility of the Health Surveillance Secretariat of the Ministry of Health. SINAN collects, processes, analyzes, and disseminates data on notifiable diseases and conditions throughout the national territory. Its coordination is divided into three spheres: national coordination carried out by the Ministry of Health, through the Health Surveillance Secretariat, state coordination by state health departments and municipal coordination by municipal health departments.^4^

The authors are certain that the systematic use of this database is the best way to evaluate and improve it, through the adequate dimensioning of health services to care for victims and awareness policies.^5^ Furthermore, the database is in the public domain, but the data available on the SINAN website are not processed, only absolute values are presented without the possibility of cross-referencing with other variables.

In Brazil, according to SINAN data, 191,653 people were victims of sexual violence between 2017 and 2021, with 140,019 reported cases of rape. A total of 169,583 cases involved female victims (88.5%), 143,341 (74.8%) of which were in the 0 to 19 years age group.^5^^,^^6^

Despite the availability of such information, few studies have been conducted to assess notifications of child sexual violence in the state. At the time of the bibliography review of the present study, the last study on the theme used data for the years 2011 to 2014 and did not assess correlations of socioeconomic indices or other types of violence with the findings.^7^

The publication of Ordinance No. 104 of 2011 of the Ministry of Health (MOH) made it compulsory to notify cases of violence, a factor that contributed to the substantial increase in the number of municipalities notifying child violence nationwide, explaining the increase in the number of cases notified over the years.^7^^,^^8^ In 2015, the MOH published a technical standard presenting a protocol of humanized attention to people in situations of sexual violence, encompassing topics such as information registration and collection of evidence.^6^ A database of sexual violence notifications is essential for the provision of comprehensive attention to people in situations of violence and the universalization of continuous surveillance systems.^4^

Given the importance of this structural problem and the scarcity of investigations on the theme, this study aimed to analyze cases of sexual violence against children and adolescents (0 at 19 years old) between 2017 and 2021 in Paraná, evaluate their geospatial distribution, and assess correlations with socioeconomic indicators. The results of the analysis may contribute to understanding the probable causes of this health problem and guiding the development of public policies aimed at reducing sexual violence against children and adolescents in the studied region.

## Methods

### Data sources and study design

This is an ecological, observational, retrospective, cross-sectional study that used secondary data obtained from SINAN. Data on sexual violence cases occurring between 2017 and 2021 in any of the 399 municipalities of Paraná State were downloaded from the online platform TABNET and analyzed using R software version 4.0.3 (The R Foundation, Austria). The data were analyzed by exploratory spatial data analysis and descriptive analysis based on measures of central tendency and dispersion (mean, median, mode, and standard deviation).

Individual notification forms (INFs), which form the structural basis of SINAN,^6^ were filtered for cases of interpersonal (sexual) violence with an age range of 0 to 19 years. The choice of this age group is in accordance with statistical and political criteria drawn up by the WHO, which defines children from 0 to 9 years old and adolescents from 10 years old to 19 years old.^9^ Subsequently, INFs were analyzed by applying additional filtering fields, such as age, sex, municipality of residence, place of occurrence (residence, school, public road, etc.), repetition (if the abuse was an isolated episode or recurrent) and identification of aggressor (life cycle of the aggressor and relationship with the victim). Because the data presented is discretized, it is not possible to assess the presence of duplicates. Data containing incomplete or ignored information were excluded.

The socioeconomic data used in the study were extracted from the Paranaense Institute for Economic and Social Development (IPARDES) database, between the years 2019 and 2021 (always using the most recent data). The independent variables were selected based on the pillars of income, health, education, and public safety: gross domestic product per capita; crime rates of threatening, swindling, rape, burglary, and theft; rate of sexual, domestic, and/or violent crimes against women; municipal development index of health; neonatal deaths; basic education development index of public elementary education, final years; basic education development index of public high schools; primary school dropout rate; and high school dropout rate.

### Georeferencing

The data collected from INFs were georeferenced using the victim's municipality of residence, and identified through a unique six-digit code standardized by the Brazilian Institute of Geography and Statistics (IBGE). For cartographic, expository, and diagrammatic purposes, a map of the political-administrative division of Paraná State and Paraná Geographic Mesoregions was used as cartographic base, available free of charge in shapefile format on the IBGE website (https://downloads.ibge.gov.br/), composed by ten mesoregions: Northwest, West Central, North Central, Pioneer North, East Central, West, Southwest, South Central, Southeast and Greater Curitiba.

### Geospatial analysis

The authors conducted exploratory spatial data analysis using GeoDA software (v1.20.0.8) and QGIS software (v3.22.4) to assess spatial autocorrelation (Moran's I). The analysis involved the calculation and visualization of local spatial association indicators (LISA) and ordinary least squares (OLS) regression.

Initially, sexual violence rates per municipality of residence of the victim were smoothed using the local empirical Bayesian estimator to mitigate extreme population effects. The authors constructed an adjacency matrix using the queen method, defining neighborhood areas based on shared borders.^10^^,^^11^ Moran's, I measure spatial autocorrelation, indicating clustered, scattered, or random patterns within spatial features and associated attributes.^12^^,^^13^ LISA was employed to identify significant local spatial associations, visualized on choropleth maps, categorizing clusters into high-high, low-low, high-low, and low-high patterns.^14^, ^15^, ^16^ The relationships between smoothed rates of sexual violence and independent variables were examined using OLS analysis, which allows for the assessment of global relationships between dependent and independent variables. This method indicates direct or inverse proportionality within a range of significance of normality (-1.96 < t < 1.96), while considering both local geographical variations and global behavioral patterns of victims.^16^ Independent variables were chosen based on indicators from the Paraná State Institute of Economic and Social Development (IPARDES), detailed before. The significance for global (Moran's I) and local (LISA) spatial autocorrelation coefficients was set at p < 0.05.^17^

### Ethical aspects

The research project complied with current ethical principles and was approved by the Human Research Ethics Committee (CAAE 57464922.0.0000.0104).

## Results

**Figure 1:**
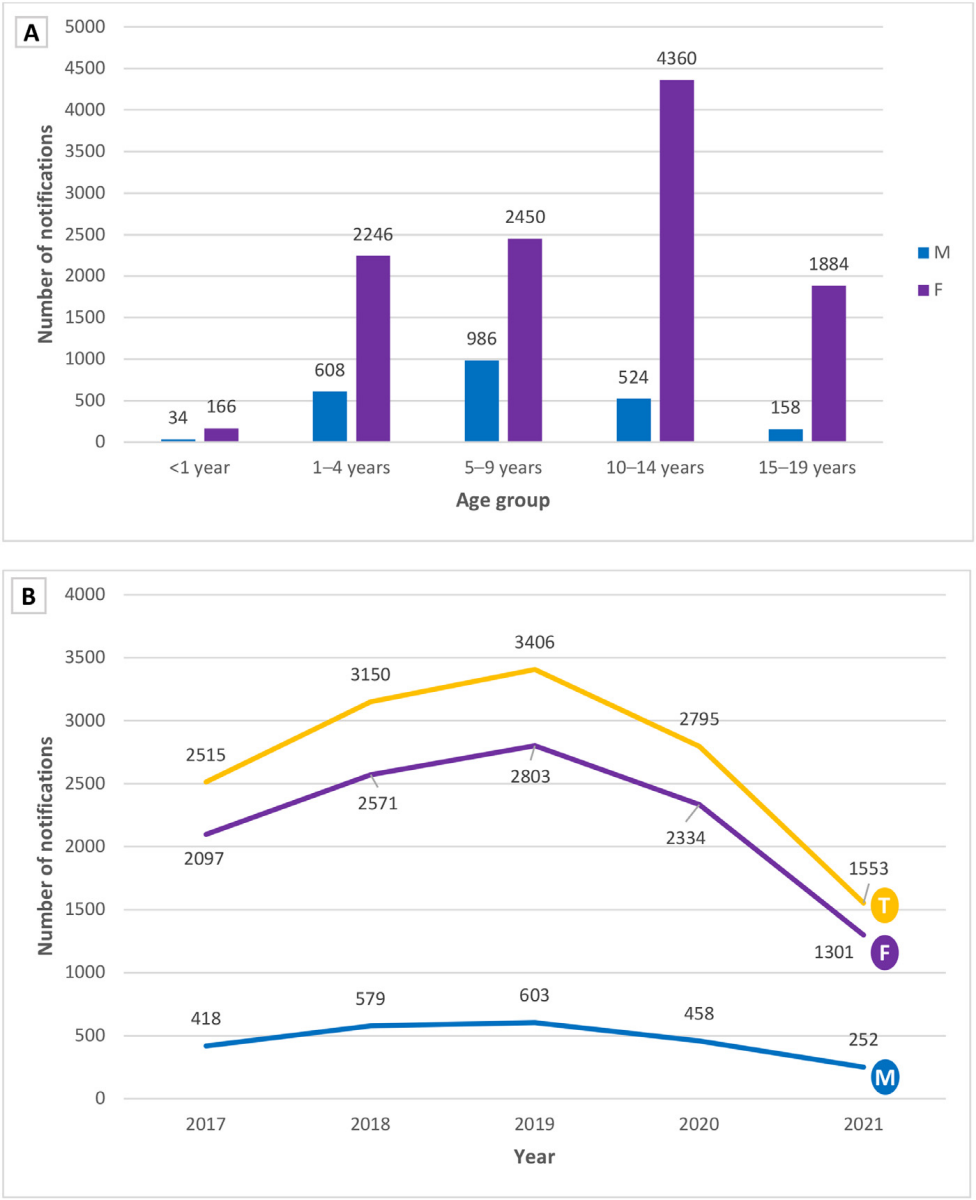
(A) Distribution of notifications of sexual violence by sex and age group and (B) annual temporal trend of notifications of sexual violence by sex in Paraná State, Brazil, from 2017 to 2021. In both figures, three notifications were discarded because the sex of victims was indeterminate. F, female; M, male; T, total.

From 2017 to 2021, 13,403 cases of sexual violence involving individuals aged 0 to 19 years were reported in Paraná State. Of these, 2,307 (17.2%) corresponded to male victims and 11,093 (82.8%) to female victims. The predominant age group of victims was 10–14 years, and a peak of cases occurred in 2019, with a decreasing trend, thereafter, as depicted in Figure 1.

The mean age of victims was 9.2 years, with a standard deviation of 4.9 years and a median of 12 years. By distinguishing sexes, it was observed that the mean age among boys was 7.5 years, with a standard deviation of 4.2 years and a median of 7 years. In girls, the mean age was 9.6 years, with a standard deviation of 4.9 years and a median of 12 years.

**Figure 2:**
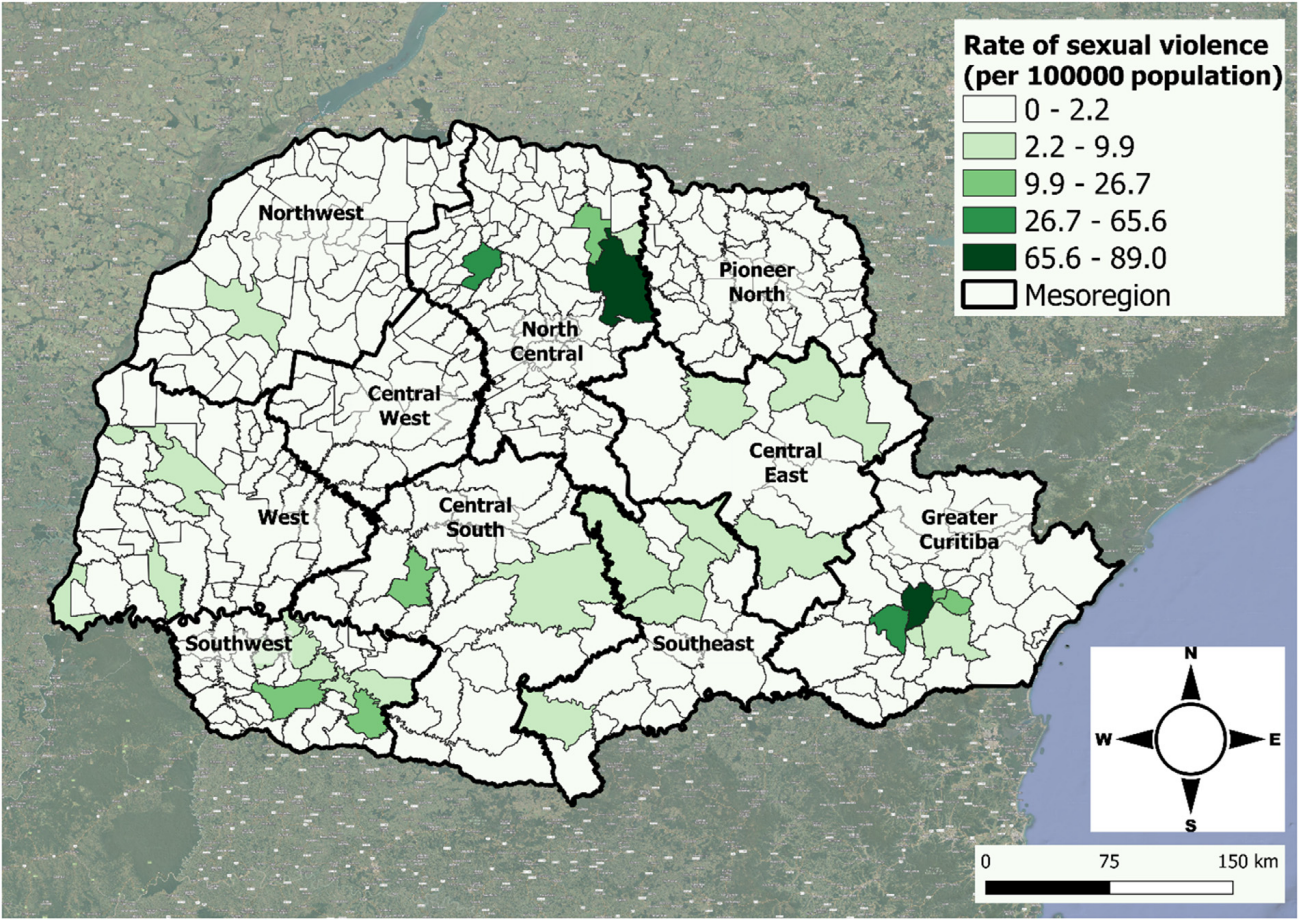
Map of the spatial distribution of mean rates of sexual violence per residence municipality in the 0–19 years age group per 100.000 population in Paraná State, Brazil, from 2017 to 2021, stratified by geographic mesoregions and smoothed through the empirical Bayesian method.

Table 1 and Figure 2 illustrate the linear and spatial distributions per municipality of the mean rate of sexual violence in Paraná State from 2017 to 2021, smoothed using the empirical Bayesian method adjusted to the 0–19 years age group and expressed per population. The highest rates of sexual violence were observed in the North Central and Greater Curitiba. Other heterogeneous foci were observed in the South Central and Southwest mesoregions.

**Table 1:** Distribution of the smoothed mean rate of sexual violence of municipalities of residence of the victims, in ascending order, in Paraná State, Brazil, from 2017 to 2021.

Rank	Municipality	SRate	Rank	Municipality	SRate
1	Curitiba	**89.0**	11	Ponta Grossa	**9.86**
2	Londrina	**84.1**	12	Coronel Vivida	**8.84**
3	Araucária	**65.5**	13	Ibiporã	**6.60**
4	Maringá	**44.9**	14	Mercedes	**6.12**
5	Piraquara	**26.7**	15	Dois Vizinhos	**5.83**
6	Pinhais	**25.6**	16	Fazenda Rio Grande	**5.69**
7	Pato Branco	**20.5**	17	Toledo	**4.93**
8	Laranjeiras do Sul	**15.3**	18	Irati	**4.79**
9	Francisco Beltrão	**14.7**	19	Telêmaco Borba	**4.74**
10	Cambé	**12.4**	20	Arapoti	**4.64**

SRate, smoothed rate of sexual violence (notifications per 100.000 population) for the 0–19 years age group.

**Figure 3:**
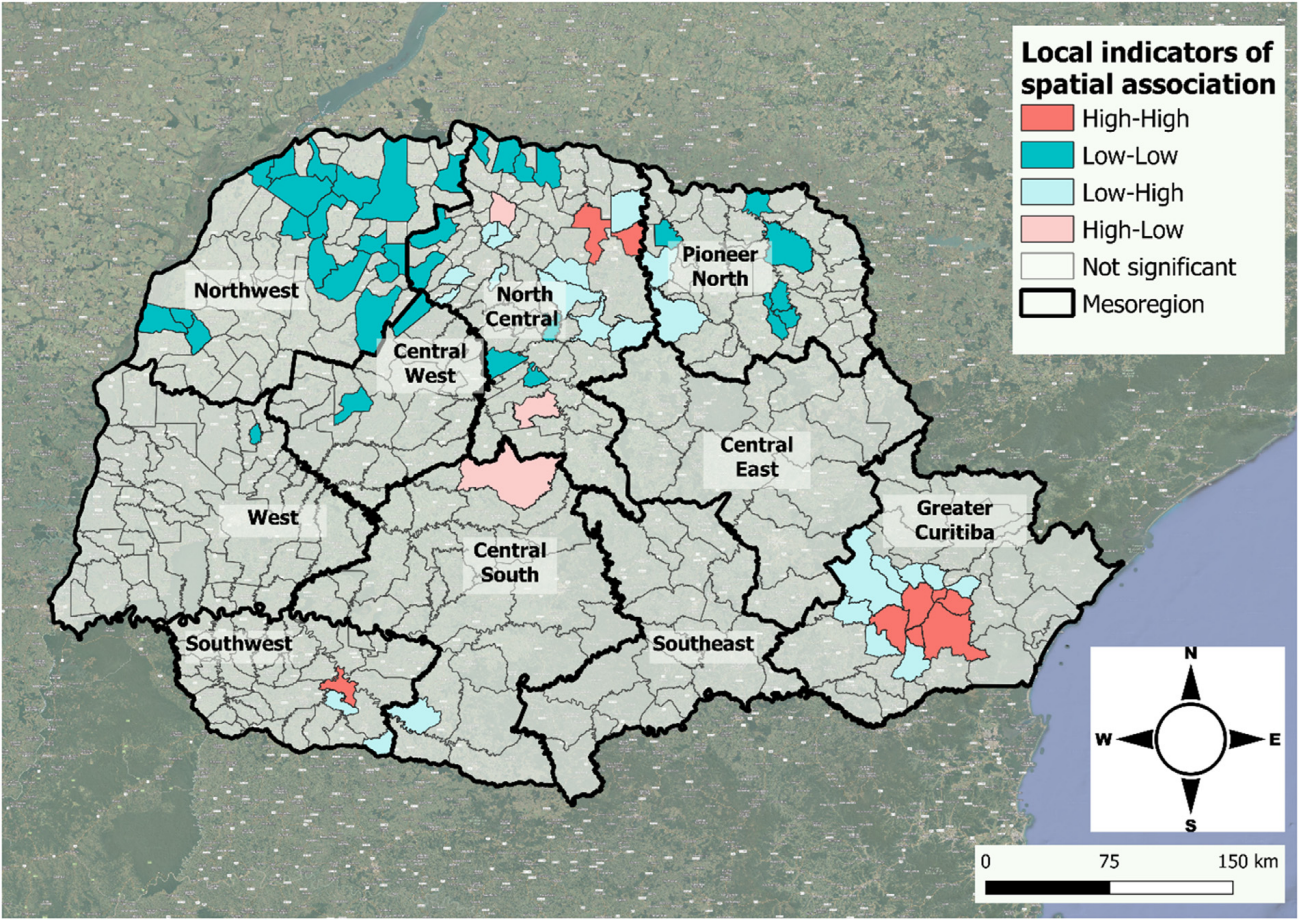
Smoothed mean rates of sexual violence in the 0–19 years age group in Paraná State, Brazil, from 2017 to 2021, stratified by geographic mesoregion.

Considering the municipalities of residence of the victims, Univariate Global Moran analysis, performed using GeoDA software, generated a Global Moran's *I* of 0.122, indicating the presence of discrete positive spatial autocorrelations between the smoothed mean rates of sexual violence in Paraná State, if most rates follow a scattered, random trend. However, by assessing LISA, the authors identified 9 high–high, 3 high–low, 36 low–low, and 23 low–high clusters, as detailed and mapped in Figure 3.

In Figure 3, it is possible to observe spatial dependence, with points of positive spatial associations (positive means), indicating that the mesoregion has neighbors with similar rates of sexual violence against children and adolescents. There was a higher number of spatial clusters in Greater Curitiba, Pioneer North, North Central, and Northwest mesoregions.

Using the OLS regression method, with the smoothed rate of sexual violence as a dependent variable, it was possible to globally test and correlate rates and independent variables (indicators). The results are shown in Supplementary Table 1. The log of the regression model had an Akaike information criterion value of −1780.17, adjusted *R*^2^ of 0.88, and a multicollinearity condition number of 25.87.

A positive correlation was observed between rates of sexual violence and gross domestic product per capita (*t* = 4.11, p < 0.05), rate of threat, swindling, rape, theft, and robbery (*t* = 7.29, p < 0.05), and rate of sexual, domestic, and/or violent crimes against women (*t* = 11.64, p < 0.05). The other variables were not statistically significant to infer a correlation.

## Discussion

According to an epidemiological bulletin of the MOH based on data from 2011 to 2018, of the 184,524 notified cases of sexual violence, 76.5% were against children and adolescents. These values underscore the possible relationship between sexual violence and the vulnerabilities.^18^^,^^19^ Furthermore, understanding that the form of the social and economic environment in which the victim is inserted seems valid, and this is a possible modified face of the reality of violence. It is also known that sexual violence can perpetuate social and economic problems.^20^

One of the problems faced in the recent past was the lack of standardization of preventive actions and reception and management of cases of sexual violence. Initially, in 2000, the Ministry of Justice drafted the National Plan for Combating Child and Adolescent Sexual Violence, and, in 2006, the Information System for Childhood and Adolescence was implemented through Ordinance No. 687. This measure allowed for continuous monitoring of cases of sexual violence against children and adolescents in guardianship councils, providing information with agility and speed to the various bodies responsible for their protection.^21^ One of the most important laws refers to the obligation of hospitals affiliated with the Unified Health System (SUS) to provide immediate care to victims of sexual violence with diagnosis and treatment of physical, medical, and psychological injuries and social support, as well as support in the registration of occurrences, referral to the Forensic Medicine Body and specialized police stations, prophylaxis (pregnancy, sexually transmitted infections), and collection of forensic samples.^22^^,^^23^

The results of this study showed that victims were mainly female and aged between 10 and 14 years, in agreement with data reported by the state protocol for attention to individuals who experienced sexual violence.^7^ Araujo and collaborators, in a previous study on sexual violence cases in Paraná State from 2011 to 2014, found a lower proportion of cases involving girls (69.6%).^24^ This discrepancy can be explained by the fact that the study period referred to the first years following the mandatory reporting of sexual violence cases.

A retrospective Spanish study investigating all types of violence (physical, neglect, sexual, and emotional) against children treated in a tertiary hospital found similar rates involving girls, but the age group differed from that observed here; in the referred study, sexual violence cases involved mainly children aged 1–5 years. Of note, only 25% of patients followed through with medical treatment and 20% with the judicial process.^25^

Analysis of cases of sexual violence according to year of notification (Figure 1B) showed a downward trend in the number of notifications in 2020 and 2021, with a reduction of 55% in 2021 in relation to 2019. According to Katz et al., in 2020, the coronavirus 2019 pandemic had a significant impact on child protection services worldwide, regardless of income. Risk factors for children increased with a concomitant temporary decrease in the reports of child maltreatment.^26^ Like the present findings, a study conducted in Santa Catarina State showed a decrease of 55.3% in the notifications registered in SINAN during the social isolation period, and there were difficulties in access to protection and assistance institutions.^27^

Geospatial analysis revealed higher smoothed rates of sexual violence in municipalities with greater absolute population densities (Curitiba, Londrina, Maringá) and their metropolitan regions, as well as in the Central and Southwestern regions of the state. In agreement with these findings, the multivariate regression spatial model showed a significant positive correlation between rates of sexual violence against children and adolescents with higher GDP per capita, higher rates of threat, swindling, rape, theft, and robbery, and higher numbers of cases of sexual, domestic, and/or violent crimes against women, which are characteristic of municipalities with higher population densities. The findings involving municipalities with high rates of sexual violence but low population density, however, are unclear and require detailed, individual analysis. Ribeiro and collaborators developed cohort studies using structural equational modeling in two Brazilian cities to analyze the effect of socioeconomic status and social support on violence involving pregnant women. Socioeconomic status had no effect on general and psychological violence, but pregnant women with low socioeconomic status had more episodes of sexual violence.^27^

Based on these findings, the authors can assist in the development of strategies to reduce the number of child sexual violence in the state of Paraná, based on regional peculiarities, which are not done to date. The public data available on sexual violence is raw and unprocessed, which makes it difficult to analyze and compare municipalities, regions, and states, as well as with other variables of interest. With the findings of the present study, the authors can help public managers plan actions to combat this severe problem in each studied region.

## Conclusion

Sexual violence against children and adolescents is a public health problem with diverse causes and identifying its relationship with other urban problems may contribute to the planning of preventive and combat strategies against sexual violence. Through this study, the authors were able to map regional disparities and their relationship with socioeconomic status and other types of violence, which can help managers outline targeted and more precise actions.

## Authors’ contributions

The conception and design of the study: Carolina Sartini Stocco, Cátia Millene Dell'Agnolo.

Acquisition of data: Carolina Sartini Stocco, Tiago Francisco Meleiro Zubiolo, Matheus Henrique Arruda Beltrame.

Analysis and interpretation of data: Carolina Sartini Stocco, Tiago Francisco Meleiro Zubiolo, Matheus Henrique Arruda Beltrame, Cátia Millene Dell'Agnolo.

Drafting the article: Carolina Sartini Stocco, Tiago Francisco Meleiro Zubiolo, Matheus Henrique Arruda Beltrame, Cátia Millene Dell'Agnolo.

Revising it critically for important intellectual content: Carolina Sartini Stocco, Cátia Millene Dell'Agnolo.

Final approval of the version to be submitted: Carolina Sartini Stocco, Tiago Francisco Meleiro Zubiolo, Matheus Henrique Arruda Beltrame, Cátia Millene Dell'Agnolo.

## Conflicts of interest

The authors declare no conflicts of interest.

## Appendix Supplementary materials

Image, application 1
