# Supplementary material for: Sexual violence against children and adolescents in Paraná State: geospatial analysis and main socioeconomic indicators
Source: J Pediatr (Rio J). 2024 May 1;100(5):498–504. doi: 10.1016/j.jped.2024.03.014 (PMC11361892; doi:10.1016/j.jped.2024.03.014)
Supplement: Supplementary file 1 [file mmc1.docx]

**JPED-D-23-00134 – Supplementary Material**

**Supplementary material Table 1** Multivariate ordinary least squares regression model.

| **Variable** | **Coefficient** | **Standard error** | ***t*-test** | **P-value** |
| --- | --- | --- | --- | --- |
| **Constant** | −0.00875439 | 0.0109929 | −0.796368 | 0.42630 |
| **GDP_PC** | 3.14002e−07 | 7.63555e−08 | 4.11236 | 0.00005 |
| **CR_T** | 0.0433362 | 0.00594372 | 7.29109 | 0.00000 |
| **SVAW_T** | 0.142625 | 0.0122524 | 11.6406 | 0.00000 |
| **IPDM_S** | 5.26851e−07 | 1.83661e−06 | 0.286861 | 0.77438 |
| **OBNEO_T** | 0.0413786 | 0.13541 | 0.305579 | 0.76010 |
| **IDEB_EF** | −0.00104593 | 0.00208489 | −0.501672 | 0.61617 |
| **IDEB_EM** | −0.000238354 | 0.00122078 | −0.195248 | 0.84530 |
| **TA_EF** | −0.458865 | 0.415374 | −1.1047 | 0.26997 |
| **TA_EM** | 0.0888818 | 0.0813057 | 1.09318 | 0.27499 |

GDP_PC, gross domestic product (GDP) per capita (R$ 1.00) [2019]; CR_T, crime rates of threatening, swindling, rape, burglary, and theft; SVAW_T, rate of sexual, domestic, and/or violent crimes against women; IPDM, IPARDES municipal development index of health [2020]; OBNEO, neonatal deaths; IDEB_EF, basic education development index of public elementary education, final years; IDEB­_EM, basic education development index of public high schools; TA_EF, primary school dropout rate (%); TA_EM, high school dropout rate (%). Data source: IPARDES, 2021.
